# Defective Efferocytosis in a Murine Model of Sjögren’s Syndrome Is Mediated by Dysfunctional Mer Tyrosine Kinase Receptor

**DOI:** 10.3390/ijms22189711

**Published:** 2021-09-08

**Authors:** Richard Witas, Astrid Rasmussen, Robert H. Scofield, Lida Radfar, Donald U. Stone, Kiely Grundahl, David Lewis, Kathy L. Sivils, Christopher J. Lessard, A. Darise Farris, Cuong Q. Nguyen

**Affiliations:** 1 Department of Infectious Diseases and Immunology, College of Veterinary Medicine, University of Florida, Gainesville, FL 32608, USA; rwitas@ufl.edu; 2 Department of Oral Biology, College of Dentistry, University of Florida, Gainesville, FL 32608, USA; 3 Genes and Human Disease Research Program, Oklahoma Medical Research Foundation, Oklahoma City, OK 73104, USA; astrid-Rasmussen@omrf.org (A.R.); kiely-grundahl@omrf.org (K.G.); chris-lessard@omrf.org (C.J.L.); 4 Arthritis and Clinical Immunology Research Program, Oklahoma Medical Research Foundation, Oklahoma City, OK 73104, USA; Hal-Scofield@omrf.org (R.H.S.); sivilsk@omrf.org (K.L.S.); darise-farris@omrf.org (A.D.F.); 5 Department of Internal Medicine, University of Oklahoma Health Sciences Center, Oklahoma City, OK 73104, USA; 6 Department of Veterans Affairs Medical Center, Oklahoma City, OK 73104, USA; 7 Department of Oral Diagnosis and Radiology, College of Dentistry, University of Oklahoma Health Sciences Center, Oklahoma City, OK 73104, USA; lida-radfar@ouhsc.edu; 8 Dean McGee Eye Institute, University of Oklahoma Health Sciences Center, Oklahoma City, OK 73104, USA; donstone13@gmail.com; 9 Department of Oral Pathology, College of Dentistry, University of Oklahoma Health Sciences Center, Oklahoma City, OK 73104, USA; david-lewis@ouhsc.edu; 10 Center of Orphaned Autoimmune Diseases, University of Florida, Gainesville, FL 32611-0880, USA

**Keywords:** autoimmunity, macrophages, efferocytosis, Mer receptor, Sjögren’s syndrome

## Abstract

Sjögren’s syndrome (SjS) is a chronic autoimmune disease primarily involving the exocrine glands in which the involvement of the innate immune system is largely uncharacterized. Mer signaling has been found to be protective in several autoimmune diseases but remains unstudied in SjS. Here, we investigated the role of Mer signaling in SjS. Mer knockout (MerKO) mice were examined for SjS disease criteria. SjS-susceptible (SjS^S^) C57BL/6.NOD-*Aec1Aec2* mice were assessed for defective Mer signaling outcomes, soluble Mer (sMer) levels, A disintegrin and metalloprotease 17 (ADAM17) activity, and Rac1 activation. In addition, SjS patient plasma samples were evaluated for sMer levels via ELISA, and sMer levels were correlated to disease manifestations. MerKO mice developed submandibular gland (SMG) lymphocytic infiltrates, SMG apoptotic cells, anti-nuclear autoantibodies (ANA), and reduced saliva flow. Mer signaling outcomes were observed to be diminished in SjS^S^ mice, as evidenced by reduced Rac1 activation in SjS^S^ mice macrophages in response to apoptotic cells and impaired efferocytosis. Increased sMer was also detected in SjS^S^ mouse sera, coinciding with higher ADAM17 activity, the enzyme responsible for cleavage and inactivation of Mer. sMer levels were elevated in patient plasma and positively correlated with focus scores, ocular staining scores, rheumatoid factors, and anti-Ro60 levels. Our data indicate that Mer plays a protective role in SjS, similar to other autoimmune diseases. Furthermore, we suggest a series of events where enhanced ADAM17 activity increases Mer inactivation and depresses Mer signaling, thus removing protection against the loss of self-tolerance and the onset of autoimmune disease in SjS^S^ mice.

## 1. Introduction

Sjögren’s syndrome (SjS) is an autoimmune disease that primarily affects women and is characterized by dry mouth and dry eye due to lymphocytic infiltration into the salivary and lacrimal glands [1]. While estimated to be the second most common autoimmune disease after rheumatoid arthritis (RA) [2], SjS remains an understudied disease, especially regarding the early phase of the disease [3,4]. Previous studies have identified aberrant gland development and accumulation of apoptotic cells in the salivary glands of both SjS patients [5] and mouse models [6]. Several factors have been implicated in this finding; specifically, the enhanced expression of Fas [7,8,9], tumor necrosis factor-related apoptosis-inducing ligand (TRAIL) [10], and Bax [11]. The aberrant apoptosis in SjS patient tissue has been reported to affect the minor salivary gland’s ductal and acinar epithelial cells [12,13]. Additionally, inflammatory cytokines tumor necrosis factor α and interferon γ were determined to induce apoptosis in a human salivary gland cell line, similarly to anti-Ro and anti-La anti-nuclear autoantibodies (ANA) that initiated apoptosis in primary salivary gland epithelial cells [14,15]. Together, data obtained from the studies of both preclinical SjS models and SjS patient samples indicate a marked propensity for apoptosis of epithelial cells coupled with increased detection of dead cells within the exocrine glands.

Under normal physiological conditions, apoptotic cells are phagocytosed and disposed of by macrophages and other phagocytes in a process termed efferocytosis. The infrequent detection of apoptotic cells in healthy tissue despite regular turnover is a testament to the importance of efferocytosis in maintaining homeostasis. Efferocytosis is an immunologically silent process characterized by suppressing inflammatory cytokines and increased molecules that resolve or limit inflammation [16]. Without timely disposal, apoptotic corpses will progress to secondary necrosis, culminating in membrane disruption and leakage of internal contents of the cell [17]. The spilled intracellular contents of the cell are interpreted by antigen-presenting cells as damage-associated molecular patterns which can induce an inflammatory and autoimmune response [18]. Indeed, it has been well established that the knockout of genes is necessary for apoptotic cell detection and clearance results in the development of autoimmunity in mice [19,20,21]. One of the most critical protein families involved in efferocytosis is the TAM receptor kinase family of Tyro3, Axl, and Mer [22]. TAMs participate in efferocytosis through a tripartite interaction involving the TAM receptor; the TAM ligands, i.e., Gas6 and protein S; and phosphatidylserine (PS) exposed on the surface of an apoptotic cell [22]. The PS-associated ligand binding activates the TAM receptor, initiating PI3K/Akt signaling and culminating in Rac1 activation [23]. Rac1 is part of the Rho GTPase family, and its activity is necessary for facilitating F-actin assembly, allowing for macrophage pseudopod formation and the uptake of dead cells [24].

Mer is the dominant receptor in homeostatic efferocytosis among the three TAM receptors and remains the best studied receptor in autoimmunity [25,26,27]. The physical interaction of membrane-bound Mer with its ligand and PS of apoptotic cells allows for optimal signaling and efferocytotic function. One mechanism that can compromise the function of Mer is the cleavage of Mer by the enzyme A disintegrin and metalloprotease 17 (ADAM17), also known as tumor necrosis factor α converting enzyme (TACE) [28,29]. ADAM17 cleaves off the N terminus of Mer, rendering the signal recognition portion of the receptor that is soluble and incapable of downstream signaling. This soluble Mer (sMer) can act as a ligand sink and impair functional Mer signaling and efferocytosis [28,29]. Cleavage-induced reduction in Mer activity through decreased active membrane-bound Mer and ligand competition with sMer has been hypothesized to contribute to autoimmune disease [30]. Despite the critical role of Mer in protecting against autoimmunity and maintaining homeostatic efferocytosis and the abundant evidence for increased apoptotic cells within the exocrine glands in SjS, the role of Mer is yet to be examined in SjS. In this study, we report that mice with systemic Mer ablation developed a SjS-like phenotype. Additionally, we discover that a SjS mouse model exhibits a loss of functional Mer mediated by ADAM17 cleavage, resulting in defective Rac1 activation, ultimately impairing efferocytosis ability. These results demonstrate a direct role for Mer in the early phase of SjS development for the first time.

## 2. Results

### 2.1. Systemic Ablation of Mer Resulted in Submandibular Gland Pathology Similar to SjS^S^ Mice

Mer is the most essential TAM receptor for macrophage efferocytosis, and defective efferocytosis can result in systemic autoimmune diseases. To determine whether systemic ablation of Mer in normal C57BL/6 results in the exocrine gland dysfunction or SjS, we utilized the B6;129-Mertk^tm1Gr^l/J (MerKO) mice. The strain was generated by introducing a neomycin cassette disrupting the last exon encoding the 3′ end of the kinase domain of Mer, preventing the expression of functional Mer proteins [31]. While Mer expression is ablated, expression levels of the other TAM receptors, i.e., Axl and Tyro3, were previously determined to be unchanged in macrophages of MerKO mice [32]. Previous studies have stated that ablation of Mer and other TAM receptors does not affect embryonic development, and TAMKO mice develop normally [31]. However, we have observed in our facility that litters of MerKO mice are strongly female skewed, with approximately 80% of our MerKO pups being born female. As a result of this male–female imbalance among MerKO mice, only female mice were used in this study. The SjS mouse model used in this study was the C57BL/6.NOD-Aec1Aec2 (SjS^S^) mouse, which has been described previously [33,34], and experiences three phases of SjS disease development coinciding approximately with 8, 15, and 24 weeks of age [35]. B6 mice were used as controls due to the B6 background of SjS^S^ mice and mixed B6/129 background of MerKO mice.

The submandibular gland (SMG) and lacrimal gland (LG) were evaluated for the presence of infiltrates, where the presence of 50 or more cells within a 4 mm^2^ area was considered positive for lymphocytic foci (LF). MerKO gland histology was compared to B6 and SjS^S^ sections. Infiltrates were not observed in the glands of young MerKO mice (8 weeks), consistent with B6 and SjS^S^ mice and previously published data [36] (Table 1). Nearly all SjS^S^ mice developed multiple SMG and LG infiltrates by 31 weeks of age (Table 1). Approximately 57% of MerKO mice displayed SMG infiltrates by 24 weeks of age, with the percentage of LF positive mouse SMG increasing with age. Conversely, only approximately 30% of MerKO mice displayed LG infiltrates, a percentage that remained constant with increasing age. Additionally, MerKO mice positive for LG infiltrates displayed fewer infiltrates than SjS^S^ mice. In summary, MerKO mice developed SMG infiltrates at similar ages to SjS^S^, with comparable percentages of positive mice and infiltrate count. However, MerKO mice featured fewer LG infiltrates with a smaller percentage of positive mice and lower focus scores than observed in SjS^S^ mice.

**Figure 1:**
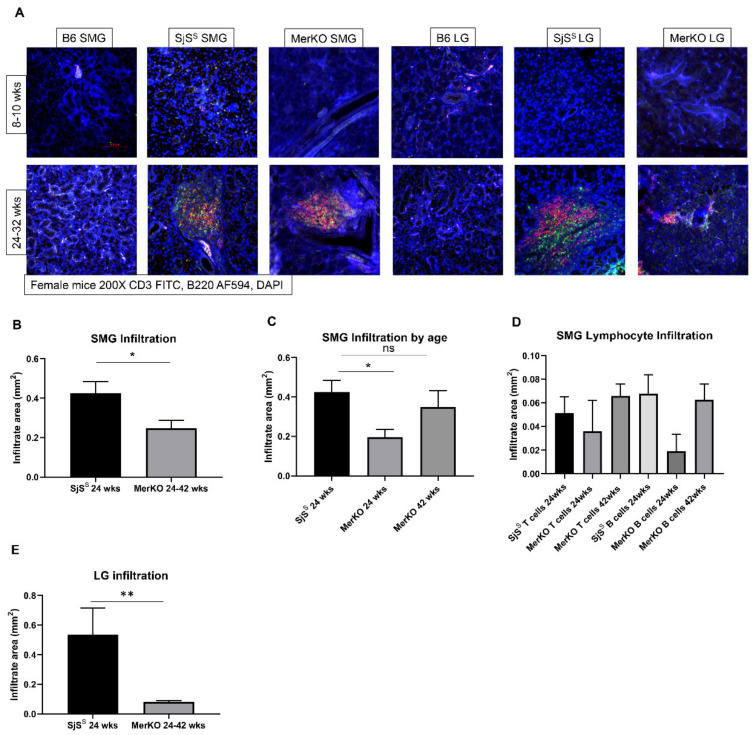
MerKO mice develop B- and T-cell infiltrates in SMG and limited LG infiltrates by 24 weeks. (**A**) Paraffin-embedded SMG and LG sections from B6, SjS^S^, and MerKO with the number of mice at each age group presented in Table 1 were stained for CD3^+^ T-cells (green), B220^+^ B-cells (red), and DAPI (blue). (**B**–**E**) The total area of infiltration, B-cells, and T-cells in the glands were determined using densitometrical analysis with ROI and threshold setting using Nikon Elements Software. The statistical significance was calculated by one-way ANOVA or two-tailed unpaired t-tests where error bars indicate SEM * *p * <  0.05, ** *p * <  0.01.

CD3^+^ T cell and B220^+^ B cell staining of SMG and LG sections revealed that the glandular infiltrates of MerKO mice were primarily composed of B and T cells, similar to SjS^S^ mice (Figure 1A). SMG infiltrates of SjS^S^ mice were larger in size than those of MerKO mice (Figure 1B). However, MerKO infiltrate size increased with age, with the infiltrate size of 42-week-old mice being significantly larger than infiltrate size in 24-week-old mice (Figure 1C). There was no significant difference in infiltrate size between the 42-week-old MerKO and 24-week-old SjS^S^ mice (Figure 1C). When determining the infiltrate composition, neither B nor T cells predominated in SMG infiltrates of MerKO mice or SjS^S^ mice (Figure 1D). Consistent with the H and E data, MerKO mice B and T-cell infiltrates in the LG were significantly reduced in size compared to SjS^S^ mice (Figure 1E). In summary, MerKO mice develop SMG B, and T-cell infiltrates increase with age and are similarly sized to SjS^S^ SMG infiltrates in aged mice. B- and T-cell infiltrates of the LG in MerKO mice are less prevalent and are smaller than those seen in SjS^S^ mice.

### 2.2. MerKO Mice Exhibit Serum Anti-Nuclear Antibodies(ANA) and Loss of Saliva Flow

**Figure 2:**
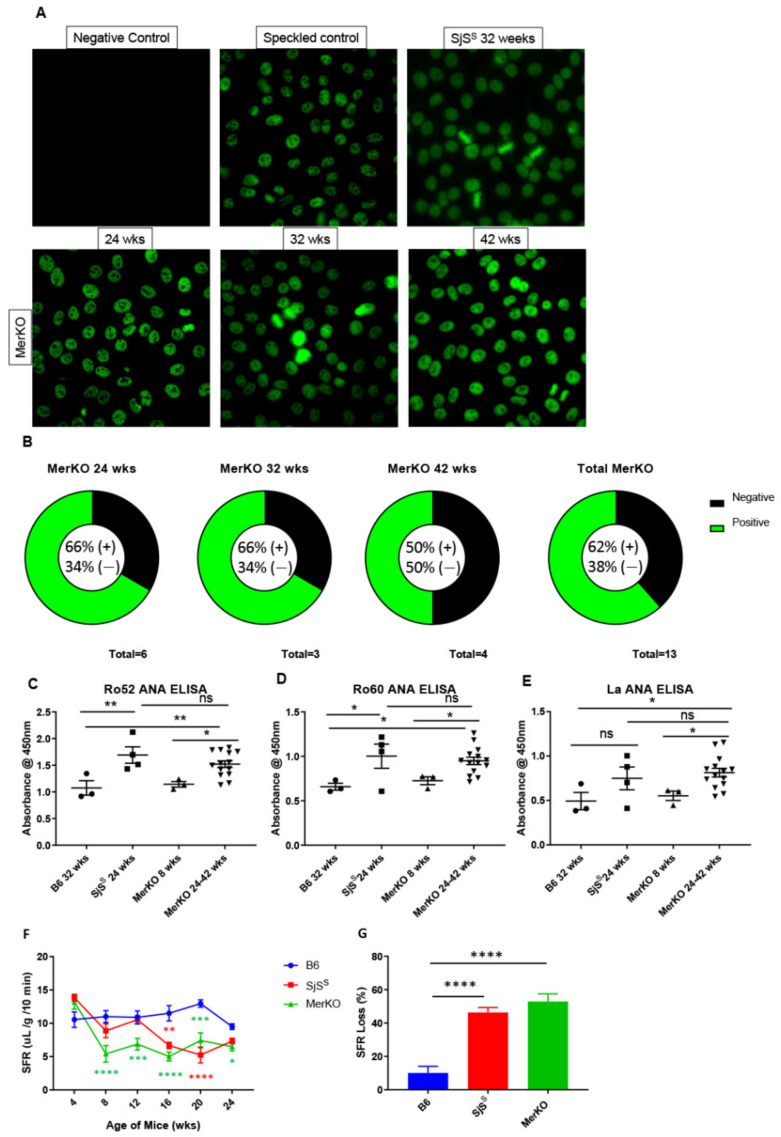
MerKO mice develop speckled ANA, anti-Ro52, Ro60, and La, and show comparable SFR loss to SjS^S^ mice with earlier onset. (**A**) Sera from MerKO (n = 13) mice was diluted 1:40 and incubated with Hep-2 slides followed by AF488 mouse secondary antibody and observation under a fluorescence microscope at 400×. (**B**) Graphs display the frequency of speckled staining patterns in MerKO sera at 24, 32, and 42 weeks of age. (**C**–**E**) B6 (n = 4), SjS^S^ (n = 4), and MerKO (n = 14) sera was diluted 1:20 and assayed for Ro52 (**C**), Ro60 (**D**), and La (**E**) antibodies via ELISA. (**F**) Saliva was collected from B6 (n = 8), SjS^S^ (n = 7), and MerKO (n = 7) mice from 4 to 24 weeks of age. Saliva data were reported as SFR in μL/gram/10 min. (**G**) The percentage of the loss of SFR between the 4-week and 24-week time points was calculated for B6, SjS^S^, and MerKO. The statistical significance was calculated by one-way ANOVA or two-tailed unpaired t-tests where error bars indicate SEM * *p*  <  0.05, ** *p*  <  0.01, *** *p*  <  0.001, and **** *p*  < 0.0001.

Seropositivity for anti-Ro ANA is a highly weighted component of the ACR/EULAR diagnostic criteria [37] and both Ro and La ANA seropositivity are included in the American European Consensus Group (AECG) criteria for SjS [38]. We compared MerKO sera to B6 and SjS^S^ mouse sera and found that 8/13 (61%) of 24-weeks-old MerKO mice and older were positive for ANA using HEp2 cell staining (Figure 2A,B). MerKO sera from mice at 24, 32, and 42 weeks was approximately 60% positive for each age group (Figure 2B). ELISA was then performed on MerKO, B6, and SjS^S^ sera samples to ascertain anti-Ro52, -Ro60, and -La ANAs. Young MerKO mice did not exhibit anti-Ro52, -Ro60, or -La ANA levels beyond what was observed in B6 controls (Figure 2C–E). This was consistent with the ANA staining where young MerKO sera were ANA negative (data not shown). Aged MerKO mice from 24–42 weeks displayed anti-Ro52, -Ro60, and -La ANA levels significantly elevated from B6 controls, and, notably, no significant difference was observed between SjS^S^ and MerKO levels of anti-Ro52, -Ro60, and -La ANAs (Figure 2C–E). Together, these data indicate that a majority of aged MerKO mice are positive for SjS-relevant ANAs at levels equivalent to SjS^S^ mice.

Finally, MerKO mice were evaluated for loss of saliva flow. MerKO mice were compared to SjS^S^ mice, which present the saliva flow reduction phenotype beginning at approximately 16 weeks of age, and B6 mice may experience a slight reduction in saliva flow with age but are largely unaffected. Surprisingly, MerKO started to exhibit a loss of saliva flow at 8 weeks of age, while SjS^S^ mice did not show a comparable loss of saliva flow until 16 weeks (Figure 2F). Furthermore, both SjS^S^ and MerKO mice exhibited relatively constant saliva flow rates (SFRs) after the initial drop, and percent loss comparing final SFRs at 24 weeks to baseline collection at 4 weeks was virtually the same between SjS^S^ and MerKO mice (Figure 2G). In summary, MerKO mice exhibited a reduction in SFR with a percent loss comparable to SjS^S^ mice; however, MerKO mice appeared to show an earlier decrease in SFR than previously seen in SjS^S^ mice.

### 2.3. Diminished Mer Function Results in Increased Apoptotic Cells and SjS^S^ Mice Display Defective Efferocytosis Signaling

SjS patients are known to develop increased apoptotic cells within their labial salivary glands [5]. SjS mouse models have also been observed to develop the same phenotype [6]. Considering that one of the primary roles of Mer is to facilitate efferocytosis by macrophages and that delayed efferocytosis is believed to contribute to autoimmunity, we evaluated the numbers of apoptotic cells in MerKO SMG. Most apoptotic cells were identified in and around areas of lymphocytic infiltration. SMG of MerKO mice showed lower levels of apoptotic cells than SjS^S^ mice, but the difference was not significant (Supplementary Figure S1A,B). Furthermore, similar to infiltrate size, the number of MerKO SMG apoptotic cells increases with age, with 24-week-old MerKO mice possessing fewer apoptotic cells than 42-week-old mice (Figure S1C).

**Figure 3:**
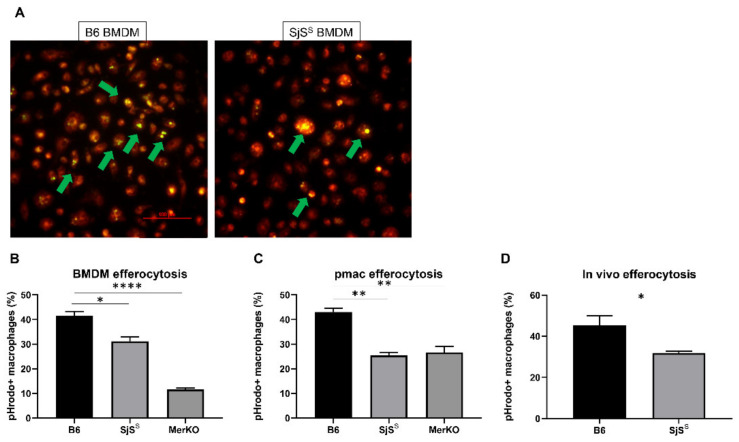
Efferocytosis is significantly reduced in SjS^S^ mice. (**A**,**B**) Bone marrow-derived macrophages (BMDM) were cultured from mice and incubated with pHrodo green (ThermoFisher) labeled apoptotic thymocytes. (**A**) B6 (n = 3) and SjS^S^ (n = 3) BMDMs were stained with CellTracker CMTPX Red (ThermoFisher) before 90-min incubation with pHrodo green-labeled apoptotic thymocytes; following washing, efferocytosis was observed using a Nikon Ti-E fluorescent microscope at 200X magnification. (**B**) B6 (n = 3), MerKO (n = 3), and SjS^S^ (n = 3) BMDMs were incubated with pHrodo-labeled apoptotic thymocytes for 90 min, and were washed, detached, and stained for macrophage markers F4/80 and CD11b. Efferocytosis was determined by flow cytometry. (**C**) Resident peritoneal macs (pmacs) were collected from B6 (n = 3), MerKO (n = 3), and SjS^S^ (n = 3) mice and were incubated with pHrodo-labeled apoptotic thymocytes for 90 min, and were washed, detached, and stained for macrophage markers F4/80 and CD11b. Efferocytosis was determined by flow cytometry. (**D**) B6 (n = 3) and SjS^S^ mice (n = 3) were injected with pHrodo-stained apoptotic cells i.p. and pmacs were collected 45 min later, stained, and analyzed for efferocytosis, as above. The statistical significance was calculated by two-tailed unpaired *t*-tests or one-way ANOVA where error bars indicate SEM * *p*  <  0.05, ** *p*  <  0.01, and **** *p*  < 0.0001.

Having observed that the complete loss of Mer in the MerKO mice results in SjS-like disease, we examined the SjS^S^ mice to determine if these mice demonstrated aberrant Mer signaling. B6 and SjS^S^ bone marrow-derived macrophages (BMDMs) were incubated with pHrodo green-labeled apoptotic thymocytes. pHrodo is weakly fluorescent at neutral pH but strongly fluorescent under acidic conditions, making it possible to observe if a pHrodo labeled cell has entered the phagolysosome of the macrophage [39]. SjS^S^ BMDMs displayed impaired efferocytosis, with fewer macrophages having successfully phagocytosed dead cells (Figure 3A,B). As expected, BMDM deficient of Mer showed significant loss of efferocytosis (Figure 3B). To further assess whether macrophages in the SjS^S^ mice display an intrinsic defect in efferocytosis, we examined apoptotic cell uptake in resident peritoneal macrophages (pmacs) ex-vivo. As presented in Figure 3C, SjS^S^ mouse pmacs also demonstrated a reduction in efferocytosis compared to control mice. The impaired efferocytosis we observed in SjS^S^ macrophages was consistent with previous reports of failure to remove apoptotic cells by macrophages in MerKO mice (Figure 3B,C) [40,41]. Impaired efferocytosis by SjS^S^ pmacs was further supported by the results of an in vivo efferocytosis assay where apoptotic cell uptake by SjS pmacs was reduced in comparison to B6 controls (Figure 3D).

### 2.4. sMer Is Elevated in SjS^S^ Mouse Sera Coinciding with the Elevated Activity of ADAM17

**Figure 4:**
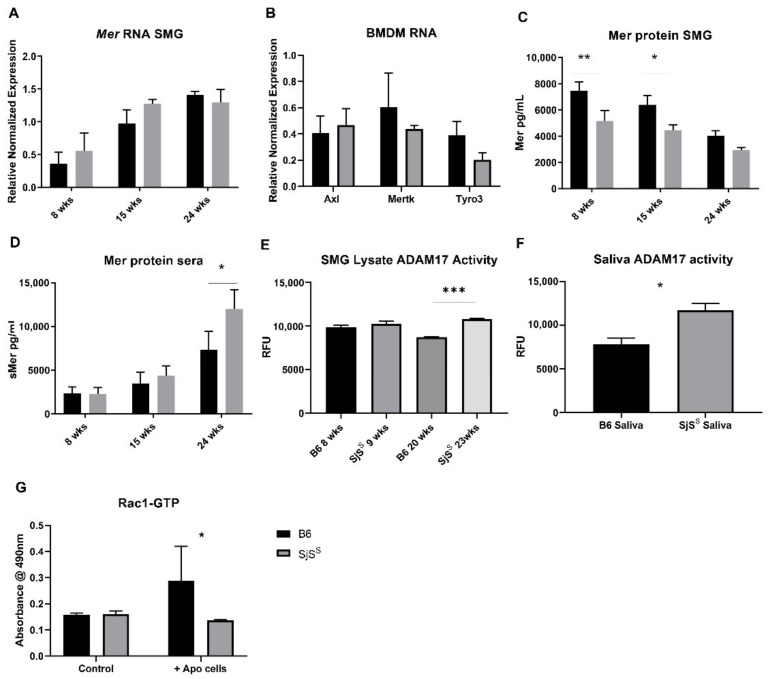
Elevated ADAM17 activity and sMer are observed in SjS^S^ mice. (**A**,**C**,**D**) Sera were collected from B6 (n = 5) and SjS^S^ mice (n = 5) at 8, 15, and 24 weeks. SMG was also excised and processed for RNA and protein using a PARIS kit. (**A**) Mer RNA expression in SMG was analyzed via RT qPCR, and expression was reported relative to GAPDH as ΔΔCq. (**B**) TAM RNA expression in BMDMs was analyzed via RT qPCR, and expression was reported relative to HPRT as ΔΔCq. (**C**) SMG protein lysate was diluted to 1 mg/mL and analyzed for Mer protein using a mouse Mer ELISA kit (RnD Systems). (**D**) Mouse sera were diluted 1:20, and Mer protein was measured using a mouse Mer ELISA kit. (**E**) SMG lysate from young (8–9 weeks) and old (20–23 weeks) B6 (n = 3) and SjS^S^ mice (n = 3) was diluted to 5 ug of protein and ADAM17 cleaving activity was measured using a SensoLyte ADAM17 activity kit. (**F**) Freshly collected mouse saliva from B6 (n = 5) and SjS^S^ mice (n = 5) (38–45 weeks) was diluted 1:100 and ADAM17 cleaving activity was measured using the same kit as (**E**). (**G**) Rac1 GLISA kit was used to measure Rac1 activation following treatment of BMDMs with apoptotic cells. The statistical significance was calculated by two-tailed unpaired *t*-tests or one-way ANOVA where error bars indicate SEM * *p * <  0.05, ** *p*  <  0.01, and *** *p*  <  0.001.

As alluded, effective Mer-mediated efferocytosis requires membrane-bound Mer, and cleavage of Mer from the cell surface inactivates the receptor, thus disabling further signaling. Therefore, we determined whether this decline in efferocytosis could result from an increase in the inactivated, soluble fraction of Mer (sMer) [28]. No differences in Mer transcript expression were detected in SMG lysate between control B6 and SjS^S^ mice at 8, 15, or 24 weeks (Figure 4A). Similarly, we observed no differences in Tyro3, Axl, or Mer transcript expression between B6 and SjS^S^ BMDMs (Figure 4B). Mer protein detected in SMG lysate, an indication of membrane-bound Mer, diminished with age in both B6 and SjS^S^ mice. However, less Mer protein was present in the SMG of SjS^S^ mice at all ages (Figure 4C). In contrast, we found sMer to be elevated in the sera, specifically at 24 weeks of age, where the difference between B6 control and SjS^S^ sMer sera levels was most pronounced (Figure 4D).

Interestingly, while B6 mice expressed higher levels of Mer in the SMG than SjS^S^ mice, there was no corresponding increase in sMer in B6 sera over SjS^S^ sera. Next, we evaluated ADAM17 activity in SjS^S^ SMG to determine whether the observed increase in sMer could be attributed to an increase in ADAM17 activity, which has been shown to cleave Mer [29]. While the difference in ADAM17 activity between young B6 and SjS^S^ mice (8–9 weeks) was minor, ADAM17 cleaving activity was significantly elevated in SjS^S^ SMG compared to B6 in older mice (20–23 weeks) (Figure 4E). Furthermore, the saliva of SjS^S^ mice possessed higher ADAM17 activity than age-matched B6 mice (Figure 4F). Together, these data indicate that SjS^S^ mice display increased sMer, and suggest that the heightened sera levels of sMer can be attributed to enhanced activity of the Mer cleaving enzyme, ADAM17.

Having observed both heightened Mer inactivation and decreased efferocytosis in SjS^S^ mice, we next investigated whether the loss of functional Mer we observed resulted in a corresponding disruption of the Mer signaling outcomes necessary for efferocytosis. The uptake of apoptotic cells by phagocytes culminates with activation of the Rho family GTPases, a process that allows for the mobilization of the cytoskeleton to form pseudopoda. Within the Rho family, Rac1 has been shown to perform a critical role in phagocyte engulfment of apoptotic cells [42]. Rac1-GTP increases following recognition of apoptotic cells by surface Mer, enabling F-actin-mediated cytoskeletal rearrangement [22]. Consequently, we investigated levels of GTP-bound Rac1 in SjS^S^ macrophages in response to apoptotic cells. As presented in Figure 4G, SjS^S^ BMDMs presented a diminished Rac1 activation in response to apoptotic cells. This finding offers a justification for the impaired efferocytosis reported in Figure 3, where apoptotic cell uptake is decreased due to decreased actin mobilization potential stemming from apoptotic cell receptor inactivation.

### 2.5. sMer Is Elevated in SjS Patient Plasma, and sMer Levels Correlate with Some Aspects of Severe Disease

**Figure 5:**
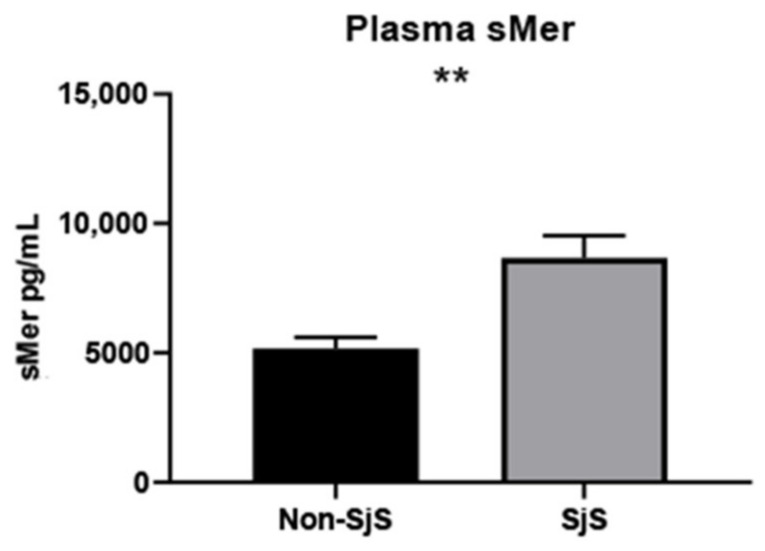
SjS patients present higher sMer levels in sera. Plasma from SjS patients (n = 15) and non-SjS patients (n = 15) was diluted 1:10 and evaluated for sMer using a human Mer ELISA kit (RnD Systems). The statistical significance was calculated by two-tailed unpaired t-tests where error bars indicate SEM, ** *p*  <  0.01.

As demonstrated, sMer is highly elevated in SjS^S^ mouse sera. We sought to determine if a similar result could be observed in SjS patients. We evaluated sMer levels in SjS patients (n = 15) and control plasma (n = 15) (Supplementary Table S1), and observed that sMer was significantly elevated in SjS patient plasma compared to controls (Figure 5). Correlation plots were constructed to determine the relationship of sMer levels to SjS disease measures. sMer levels were found to be correlated to salivary gland lymphocytic infiltration (i.e., focus score (FS), van Bijsterveld (vBS), ocular staining score, rheumatoid factor (RF) levels, and Ro60 ANA positivity (Table 2). No correlation was found between sMer levels and anti-Ro52, anti-La, ANA levels, IgG levels, EULAR Sjögren’s syndrome disease activity index (ESSDAI), Schirmer’s test results, whole unstimulated salivary flow (WUSF), erythrocyte sedimentation rates (ESR), and age (Table 2). In summary, we demonstrate that sMer, which hinders Mer signaling and efferocytosis, is elevated in the plasma of SjS patients, and elevated sMer is associated with several classical clinical symptoms.

## 3. Discussion

Cellular turnover of the exocrine glands is a well-regulated process of tissue homeostasis. Apoptosis and cell renewal are essential for healthy gland development. Excessive apoptosis and defective clearance of cellular debris play a crucial role in autoimmunity. SjS is a complex autoimmune disease with a multifactorial etiology. Unregulated apoptosis coupled with impaired clearance may contribute to SjS etiology by triggering inflammation and amplifying the autoimmune response with the spread of autoantigens. One effective mechanism regulating apoptotic clearance is the TAM pathway, specifically the receptor Mer [43]. Previous studies investigating Mer in other autoimmune diseases have suggested that the TAM pathway is critical for maintaining self-tolerance and preventing autoimmunity [44]. This study sought to investigate the relationship between Mer, the most studied TAM in autoimmunity, and SjS. The results demonstrate that Mer plays a critical protective role in SjS. This conclusion is based upon both the finding that systemic Mer ablation in mice results in SjS-like disease and the observation that a SjS mouse model exhibits decreased Mer signaling and efferocytosis due to enhanced cleavage and inactivation of Mer.

We first sought to characterize how the loss of Mer signaling contributes to SjS by investigating SjS disease criteria in the MerKO mouse. MerKO mice primarily experience SMG pathology with minimal lacrimal gland involvement, as evidenced by the presence of B- and T-cell infiltrates in the SMG of comparable size and number to SjS^S^ mice. MerKO mice also developed apoptotic cells in the SMG similar to what was detected in SjS^S^ mice. While MerKO mice are known to experience elevated apoptotic cells in some tissues, this is the first evidence of this phenotype occurring in the SMG, analogous to what is observed in SjS. Sera from most MerKO mice possessed a speckled ANA staining pattern, and levels of Ro52, Ro60, and La ANAs were comparable to SjS^S^ mice. While elevated anti-Ro antibodies in the sera alone are not diagnostic for SjS according to clinical criteria, in conjunction with other criteria, i.e., SMG infiltration, the presence of these antibodies supports the existence of a SjS phenotype in MerKO mice. MerKO mice demonstrated a comparable loss in saliva flow rate to SjS^S^ mice. The early onset of saliva loss in MerKO mice warrants further investigation, as it precedes infiltrate formation, accumulation of apoptotic cells, and appearance of ANAs. It has previously been established that hyposalivation is not solely dependent on lymphocytic infiltration [45]. B cells have also been implicated in hyposalivation by producing autoantibodies obstructing receptors required for salivation [46]. However, investigating the inflammatory cytokine levels in the SMG of young MerKO mice may offer a more clarifying explanation for early-onset hyposalivation, considering the lack of other autoantibodies in mice of this age [45]. The lack of LG pathology in contrast to SMG pathology was another unexpected finding in MerKO mice. This discovery suggests that severe LG pathology is not induced by ablated Mer signaling alone and requires additional pathogenic stimuli. Susceptibility to apoptotic signals is better understood in the salivary gland epithelial cells (SGECs) [47] than in the LG, so it is possible that the contrasting responses to defective Mer signaling in SG and LG could be due to differences in propensity for apoptosis between these two tissues.

Having observed that the loss of Mer is sufficient for the development of SjS-like disease, we evaluated Mer signaling in SjS^S^ mice. We found that both BMDM and resident pmacs from SjS^S^ mice performed decreased efferocytosis compared to B6 controls. These results complemented previous investigations of SjS patient monocyte-derived macrophages and NOD mouse BMDMs [48,49]. Monocyte-derived macrophages from SjS patients were found to perform decreased efferocytosis and display an inflammatory cytokine profile upon exposure to apoptotic cells. Both of these features resisted treatment with the anti-inflammatory vasoactive intestinal peptide [49]. The inflammatory response reported in SjS macrophages following efferocytosis was particularly relevant to our own findings, as Mer signaling performs a dual role in facilitating efferocytosis and dampening inflammation [50]. NOD pmacs and BMDMs were described to perform proper phagocytosis of foreign bodies but impaired efferocytosis of thymocytes, a feature the authors attributed to perturbed expression or turnover of apoptotic cell recognition receptors [48]. Additionally, a study using elicited pmacs from the atherosclerosis model, *Aath4a^DBA/DBA^*, with reduced Mer expression reported that efferocytotic capacity was directly proportional to macrophage Mer expression [51]. These data support the notion that the diminished efferocytosis we observed could be attributable to altered surface Mer expression in SjS^S^ mice.

We next explored how Mer signaling could be reduced in SjS^S^ mice by measuring functional membrane-bound and nonfunctional soluble Mer levels. SjS^S^ mice SMG contained less Mer protein, and SjS^S^ mouse sera had higher levels of sMer in sera, possibly due to increased ADAM17 activity. ADAM17 expression has been reported to be significantly higher in SjS labial SG biopsies compared to non-SjS controls [52]. Our investigations found increased ADAM17 activity in the SMG of SjS^S^ mice at 23 weeks of age compared to controls. Furthermore, another study found expression of the ADAM17 activating enzyme, Furin, which is elevated in SjS patient plasma and PBMCs [53], suggesting a possible mechanism in SjS where increased Furin levels stimulate higher ADAM17 activity promoting Mer cleavage. Interestingly, treatment of SGECs with anti-Ro and anti-La ANAs was also found to increase ADAM17 expression [54], allowing for an alternative mechanism driving Mer cleavage. Treatment of SjS^S^ BMDMs with apoptotic cells resulted in reduced Rac1 activation compared to controls, indicating an impaired downstream aspect of Mer signaling directly facilitating efferocytosis. Previous investigation has demonstrated that depletion of Mer reduces Rac1 activity, supporting a model where decreased functional Mer levels in SjS^S^ mice limits actin mobilizing ability and efferocytosis [55]. Elevated sMer levels were also detected in SjS patient plasma and correlated with FS, ocular staining score, RF, and anti-Ro60 levels. The correlations between sMer, FS, RF, and anti-Ro60 can be rationalized by reduced functional Mer, impairing apoptotic uptake and allowing for self-antigen leakage, inflammation, and reaction against self-proteins, as described in SLE [56]. This concept is supported by our observation of both elevated apoptotic cells and lymphocytic infiltrates within MerKO SMG. Conversely, the correlation between sMer and ocular staining score was unexpected because of the minor LG infiltration seen in MerKO mice. However, one must keep in mind that the limited genetic diversity within a single mouse model precludes representation of all the possible heterogeneous presentations of SjS [35]; this finding may perhaps indicate that human LG epithelial cells are more susceptible to the consequences of the loss of Mer signaling than those of mice. It is also important to consider that the small patient pool examined here may impede the detection of relationships between sMer and other disease manifestations. This may have been the case in the correlation with ESSDAI, as many of the SjS patients in this study had low ESSDAI scores (average 1.13). While the lack of correlation between ESSDAI and sMer levels was perplexing and could be due to the small sample size, the correlations we did observe regarding Ro60 and SG infiltration reinforce the association between Mer inactivation and the development of autoimmunity in SjS. Interestingly, a previous study also found that sMer was elevated in SjS patient plasma and reported that sMer levels correlated with IgG levels, ESR, and ESSDAI, and that sMer levels were higher in Ro/La ANA-positive patients [57].

## 4. Conclusions

**Figure 6:**
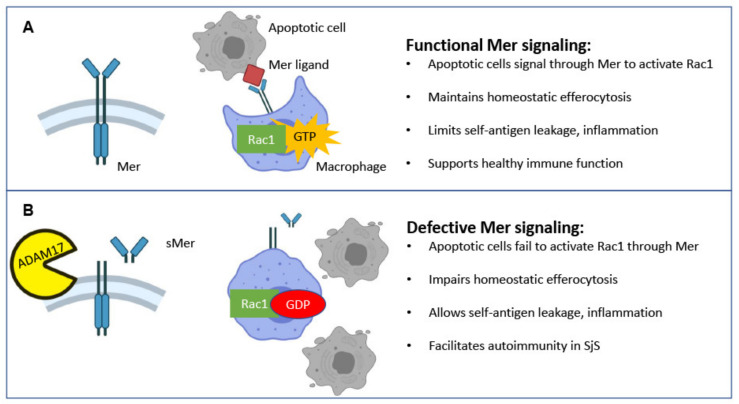
(**A**) Under healthy conditions, membrane-bound Mer on the surface of macrophages is activated by apoptotic cells, stimulating Rac1 activation and actin mobilization necessary for the uptake of apoptotic cells. The removal of apoptotic cells plays a critical role in maintaining self-tolerance by preventing the leakage of self-antigen from secondary necrotic cells. (**B**) The impaired efferocytosis mediates the autoimmune process of SjS with reduced Rac1 activation. This process is attributed to defective Mer signaling and excessive cleavage by activation of ADAM17 function.

Due to the insidious nature of SjS and the lack of early identifying biomarkers, understanding early disease events and contributions by the innate immune system has proven difficult. Here, as illustrated in Figure 6, we propose a sequence of events where elevated activation of ADAM17 leads to enhanced cleavage of Mer into sMer and the lack of functional Mer receptor depresses Mer signaling. This deficiency inhibits apoptotic cell clearance, thereby establishing an environment supportive for the development of autoimmune disease. This concept is supported by our data indicating that mice lacking Mer expression experience a disease phenotype comparable to what we observe in SjS^S^ mice. However, it remains to be seen if sMer levels are only related to disease severity or if sMer acts in a pathogenic manner, as suggested in SLE [58]. Obvious next steps to pursue include investigating how closely AxlKO and Tyro3KO mice resemble MerKO mice in SjS diagnostic criteria. Additionally, evaluating the extent that Mer signaling is compromised in SjS^S^ mice by enhanced Mer cleavage, especially regarding inflammation regulation, remains to be performed. Furthermore, it would be illustrative to evaluate the effect of overexpression of Mer, either systemically or specifically in SMG of SjS^S^ mice on disease severity.

## 5. Materials and Methods

### 5.1. Mice

C57BL/6 (B6), B6J.NOD/ShiLtJ-*Aec1Aec2* (B6.NOD-*Aec1Aec*2), and B6;129-*Mertk^tm1Grl^*/J (MerKO) mice were bred and maintained under specific pathogen-free conditions in the animal facility of Animal Care Services at the University of Florida. All animals were maintained on a 12-h light–dark schedule and were provided with food and water *ad libitum*. Mice were anesthetized with isoflurane and euthanized by cervical dislocation, and their organs and tissues were freshly harvested for analysis. All mice included in this study were female. The University of Florida’s Institutional Animal Care approved all protocols respective to breeding and the use of animals described herein. The experimental methods were carried out in accordance with the appropriate approvals and relevant guidelines.

### 5.2. Patients

The human subjects were participants in a large cohort of sicca patients evaluated in the Sjögren’s Research Clinic at Oklahoma Medical Research Foundation (OMRF) [59]. All the tests necessary for classification by AECG [38] and ACR-EULAR [37] criteria were performed in addition to the collection of detailed clinical and serological measures, as previously described [59]. The definition of SjS case or control was based on the AECG classification criteria. The Institutional Review Board of each institution approved all procedures, and each participant provided written informed consent prior to entering the study. The study was conducted in accordance with current regulations protecting human subjects participating in research, HIPAA, and the Declaration of Helsinki.

### 5.3. Histological Grading

Submandibular and lacrimal glands from B6, SjS^S^, and MerKO mice (8–42 weeks of age) were immediately collected and fixed by immersion in 10% phosphate-buffered formalin for at least 24 h. Fixed tissues were embedded in paraffin and sectioned at a thickness of 5 μm. Hematoxylin and eosin (H and E) stained sections were observed at 200× magnification using a Nikon Eclipse Ti-E inverted microscope. Two histological sections per gland per mouse were examined, and lymphocytic infiltrations were defined as aggregates of >50 leukocytes. Three independent pathologists performed histological examination in a blinded manner.

### 5.4. Immunofluorescent Staining for CD3 and B220

Paraffin-embedded sections from submandibular and lacrimal gland tissue from B6, SjS^S^, and MerKO mice (8 to 42 weeks of age) were deparaffinized, rehydrated, and unmasked by immersion in Trilogy (Cell Marque, Rocklin, CA, USA) solution, and heated in a pressure cooker for 15 min. Staining procedures for CD3+ T-cells and B220+ B-cells were followed, as described previously (1). Stained sections were visualized at 200× magnification using a Nikon Ti-E fluorescent microscope. Nikon NIS-Elements software (Nikon, Minato City, Tokyo, Japan) was used to determine the composition and size of infiltrates based upon the region of interest function (ROI), as previously described by our group (2).

### 5.5. Terminal Deoxynucleotidyl Transferase dUTP Nick end Labeling (TUNEL) Staining for Apoptotic Cells

Paraffin-embedded tissues of the submandibular glands from B6, SjS^S^, and MerKO mice (8 to 42 weeks of age) were deparaffinized and dehydrated by pressure-cooking in Trilogy (Cell Marque, Rocklin, CA, USA), as mentioned above. Sections were washed three times for five minutes with phosphate-buffered saline (PBS) with Tween-20 (PBS-T), followed by treatment with proteinase K for 30 min. The sections were washed again with PBS-T. The positive control was incubated with 0.5 mg of DNase I (Sigma Aldrich, Saint Louis, MO, USA) for 10 min. Next, sections were incubated with terminal deoxynucleotidyl transferase (TdT) and Biotin-11-dUTP in Tris cacodylate buffer for 1 h at 37  °C. The reaction was halted by incubating slides with sodium citrate buffer for 10 min. Apoptotic cells were then fluorescently labeled by incubating slides for one hour with AF488 streptavidin, followed by mounting using Vectashield DAPI-mounting medium. Stained slides were observed at 400× magnification on a Nikon Ti-E fluorescent microscope using an exposure of 200 milliseconds. Apoptotic cells were counted within each gland, and the total number of apoptotic cells detected per gland were reported. Threshold intensities were maintained across samples for consistency.

### 5.6. Detection of Anti-Nuclear Antibodies (ANA) from Sera

The HEp-2 ANA kit (Inova Diagnostics, Inc., San Diego, CA, USA) was used to detect ANA in sera from SjS^S^ and MerKO mice (24 to 42 weeks of age), according to the manufacturer’s protocol, as our group has previously described (2). Stained Hep-2 slides were observed at 400× magnification on a Nikon Ti-E fluorescent microscope using an exposure of 200 milliseconds. Anti-Ro52, anti-R60, and anti-La were detected from mouse sera using ELISA, as we have previously described (1). Recombinant human Ro52/SS-A (12700), Ro60/SS-A (15500), and La/SS-B (12800, Diarect, Freiburg, Germany) were diluted to a concentration of 0.6 ug/mL in carbonate buffer pH 9.6 and added to the individual wells of a 96-well plate overnight at 4 °C. Next, the wells were washed with PBS-T and then blocked in 5% bovine serum albumin in PBS at pH 7.4 overnight in 4 °C. Sera samples were diluted 1:20 in 200 uL and added into each well in duplicate. Sera samples were incubated in the plate at room temperature for 2 h. The wells were then washed again with PBS-T, incubated with a 1:10,000 dilution of anti-mouse IgG (SC-2005, Santa Cruz Biotech, Santa Cruz, CA, USA), and conjugated to horseradish peroxidase (HRP) in PBS for 2 h at room temperature. After washing the plate with PBS-T, the wells were treated with 100 uL of TMB substrate solution (00-4201-56, eBioscience, San Diego, CA, USA) for 30 min with shaking and followed by treatment with 3N HCl to stop the reaction. Absorbance was read at 450 nm using a Tecan Infinite M200 Pro spectrophotometric plate reader. The data were reported as relative absorbance.

ANA staining was initially performed at 1:40 sera dilution. Additional dilutions were performed to determine antibody titer. The speckled staining pattern was visible in MerKO sera samples up to a 1:120 dilution, suggesting a robust ANA presence (Figure S2).

### 5.7. Saliva Collection

B6, SjS^S^, and MerKO mice (8 to 24 weeks of age) were weighed and given a 100-μL intraperitoneal (i.p.) injection of isoproterenol (0.2 mg/1 mL of PBS) and pilocarpine (0.05 mg/1 mL of PBS), a cocktail known to stimulate saliva flow. For 10 min, saliva was collected from the oral cavity of restrained mice using a micropipette, with the collection beginning one minute after injection. The volume of saliva produced by each mouse was measured by pipette. The saliva flow rate was determined by dividing the volume of saliva (μL) collected during a 10-min period by the weight of the mouse (grams).

### 5.8. Bone Marrow-Derived Macrophages (BMDM) Isolation

BMDMs were grown, as described previously [60]. Briefly, 7–12-week-old B6, MerKO, and SjS^S^ mice were euthanized, and bone marrow cells were collected by flushing the femurs and tibias with RPMI (Lonza, Allendale, NJ, USA) complete media containing 10% FBS, 1% penicillin/ streptomycin, 2 mM of L-glutamine, 0.05 mM of β-mercaptoethanol supplemented with 20% L929 supernatant containing M-CSF and L929 media preparation, as described in [60]. Cells for each mouse were filtered through a 70-μm cell strainer and plated in untreated Petri dishes with 5 million cells per dish. Cells were maintained in a 37 °C incubator for seven days with 50% media changes performed every two days. When differentiation was completed after seven days, macrophages were detached from plates by 15-min incubation with Accutase (Innovative Cell Technologies, San Diego, CA, USA) on ice. Cells were counted and redistributed to 24-well plates with 3 × 10^5^ cells per well, and L929 supplementation was reduced to 5% 24 h before treatment.

### 5.9. Rac1 GLISA Assay

BMDMs were differentiated, as described previously, and were treated with apoptotic cells for 30 min. BMDMs were washed 3 times in ice-cold PBS and harvested, according to the Rac1 GLISA activation assay manufacturer protocol (Cytoskeleton, Denver, CO, USA). Briefly, BMDMs were treated with lysis buffer, scraped, and snap-frozen in liquid nitrogen until used. Absorbance was read at 490 nm using a Tecan plate reader. The data were reported as relative absorbance.

### 5.10. Peritoneal Macrophage (Pmacs) Isolation

B6, MerKO, and SjS^S^ resident pmacs were collected, as described previously [61]. Briefly, 7–12-week-old mice were euthanized, and mouse peritoneal cavities were washed 3× with 10 mL of ice-cold PBS with 10% FBS. Peritoneal cells were depleted of B cells through negative selection. B cells were labeled using a PE-conjugated anti-B220 antibody (553090 BD Biosciences San Jose, CA) for 30 min on ice followed by a wash and anti-PE magnetic beads (130 058 801 MACs Miltenyl Biotech Bergisch Gladbach, Germany). After 20 min of incubation on ice with magnetic beads, the cells were moved to a DynaMag-15 magnet (Invitrogen, Waltham, MA, USA), and the macrophage containing supernatant was collected. Macrophages were counted and plated on 24-well plates 3 × 10^5^ cells per plate in RPMI complete media. Cells were assayed the following day.

### 5.11. Thymocyte Collection, Apoptosis, and Staining

Thymi were collected from 7–12-week-old mice and cut into small pieces using scissors. Thymi were incubated in an enzymatic solution containing RPMI, 1 mg/mL of collagenase IV (Sigma Aldrich), and 1 mg/mL of DNase I (Signma Aldrich) at 37 °C for 20 min. The supernatant containing thymocytes was collected on ice in RPMI with 10% FBS. The new enzyme solution was added to thymus tissue and agitated using a 5-mL pipette before incubating for an additional 15 min. The process was repeated with an 18-G needle for mechanical agitation. The process was repeated using a 25-G needle, and then the supernatant was passed through a 70-μm cell strainer before the cells were washed and counted. Thymocytes were rendered apoptotic by a 4-h incubation with 2 μM of dexamethasone (Tocris, Bristol, UK) at 37 °C in RPMI supplemented with 1% FBS. Thymocytes were washed twice in PBS and were determined to be greater than 90% apoptotic by Trypan Blue staining. Apoptotic thymocytes were stained with pHrodo green (P35373 ThermoFisher, Waltham, MA, USA) and washed the following staining according to manufacturer instructions.

### 5.12. In Vitro (BMDM) and Ex Vivo (Pmac) Efferocytosis Assay

Stained syngeneic apoptotic thymocytes were incubated with macrophages in a 5:1 ratio for 90 min at 37 °C followed by three washes to remove apoptotic cells. Macrophages were detached from plates via incubation in Accutase for 15 min and gentle scraping. Macrophages were washed and stained for the macrophage markers F4/80 (123110 Biolegend, San Diego, CA, USA) and CD11b (101243 Biolegend) for 30 min on ice, followed by washing and observation of macrophages on a BD LSR Fortessa flow cytometer (BD Biosciences). BMDMs or pmacs were gated on singlets, FSC, SSC, and F4/80 CD11b double positivity. Flow analysis was performed using Flowjo v10 software (Flowjo Ashland, OR, USA). Alternatively, efferocytosis was observed via microscopy. BMDMs were stained with CellTracker CMTPX Red (ThermoFisher) via a 30-min incubation followed by two washes. Apoptotic pHrodo stained thymocytes were incubated with BMDMs in a 5:1 ratio for 90 min at 37 °C. BMDMs were washed three times and immediately observed using a Nikon Ti-E fluorescent microscope.

### 5.13. In Vivo Efferocytosis Assay

Apoptotic thymocytes were generated and stained with pHrodo as described above. Eight-week-old B6 (n = 3) or SjS^S^ mice (n = 3) were injected i.p. with 4 × 10^6^ stained apoptotic cells. Forty-five minutes after injection, pmacs were collected according to pmac isolation protocol described above and stained for flow cytometry in the same manner as the in vitro efferocytosis assay.

### 5.14. Detection of Mer from Mouse Tissue, Sera, and Human Plasma

The ELISA assay for mouse Mer was performed, as described by the manufacturer of the Mouse Mer ELISA kit (DY591 RnD Systems, Minneapolis, MN, USA). The total human Mer ELISA kit (DYC891 RnD Systems) was used for the detection of Mer from human samples. Briefly, 96-well plates were coated overnight with the Mer capture antibody diluted to 8 ug/mL in PBS at room temperature. Plates were washed the following day with wash solution and blocked for 1 h with reagent diluent. Both wash and reagent diluent were from ancillary reagent kit DY008 (RnD Systems, Minneapolis, MN, USA). Plates were washed again before mouse sera samples from B6 and SjS^S^ mice (8, 15, and 24 weeks of age) were diluted to 1:20, and mouse submandibular gland lysate samples were diluted to 1 mg/mL of protein in reagent diluent to a volume of 100 uL and incubated in the plates for two hours at room temperature. Alternatively, human plasma samples were diluted 1:10 under the same conditions. Plates were washed again, and biotinylated detection antibody was added to the plates at a concentration of 200 ng/mL for two hours at room temperature. Plates were washed and incubated with a 1:200 dilution of streptavidin-HRP for 20 min in the dark at room temperature. Plates were washed, and 100 uL of substrate solution was added to the plates for 20 min in the dark before the reaction was stopped by adding 50 uL of stop solution to each well. Optical density for each well was measured at 570 nm, and this value was subtracted from the reading at 450 nm using a Tecan Infinite M200 Pro spectrophotometric plate reader (Tecan, Mannedorf, Switzerland). Sample Mer concentration was interpolated from a seven-point standard curve of Mer standard, and values were reported as pg/mL.

### 5.15. RT qPCR for Mer from Mouse Submandibular Glands and BMDM

B6 and SjS^S^ mice were euthanized at 8, 15, or 24 weeks. Submandibular glands were removed and stored in RNAlater (Thermofisher) at −20 °C until required. RNA and protein were isolated from SMG tissue stored in RNAlater using a ThermoFisher PARIS (protein and RNA isolation) kit, according to manufacturer protocol. A Biorad RT qPCR 1 step kit (Bio-Rad, Hercules, CA, USA) was used to measure the expression of *mer* with *gapdh* as a reference gene according to manufacturer protocol. Alternatively, BMDM RNA was evaluated. *mer* f: 5′-ACC TCC ACA CCT TCC TGT TA-3′, mer r: 5′-CGT GGA GAA GGT AGT CGT ACA TCT-3′, *gapdh* f: 5′- TCC CAC TCT TCC ACC TTC GA -3′, *gapdh* r: 5′- AGT TGG GAT AGG GCC TCT CTT -3′, *axl* f: 5′-ATG CCA GTC AAG TGG ATT GCT-3′, *axl* r: 5′-CAC ACA TCG CTC TTG CTG GT-3′, *tyro3* f: 5′-CAT TCC AGA GCA GCA GTT CAC-3′, *tyro3* r: 5′-CCA CAC ACA CTG TCA TGT CCT-3′, *hprt* f: 5′-AGT GTT GGA TAC AGG CCA GAC-3′, *hprt* r: 5′-CGT GAT TCA AAT CCC TGA AGT-3′. Analysis of RT qPCR data was performed using CFX Maestro software (Bio-Rad), and expression was reported as ΔΔCq.

### 5.16. ADAM17 Activity Assay

Freshly collected B6 and SjS^S^ (38–45 weeks of age) mouse saliva, collected as above, was tested for ADAM17 activity using a SensoLyte 520 fluorimetric ADAM17 activity kit (AnaSpec, Fremont, CA, USA). Briefly, mouse saliva was diluted 1:100 in an assay buffer provided within the kit, and 50 uL of each sample was added to a black 96-well plate in duplicate. Alternatively, 5 ug of SMG lysate homogenized as above from B6 and SjS^S^ mice (8–9, or 20–23 weeks of age) was diluted in a sample buffer and tested in duplicate. An equal volume of ADAM17 substrate was added to each well of the sample. The plate was shaken for 30 s and incubated in the dark for 30 min at room temperature. Next, 50 uL of STOP solution was added to each well. The plate was shaken again for 30 s, and fluorescence was read using a M5 spectramax microplate reader at 490/520 nm (Molecular Devices, San Jose, CA, USA). Finally, fluorescence was reported as relative fluorescent units (RFUs).

### 5.17. Statistical Analyses

Statistical evaluations were determined using one-tailed Mann–Whitney *t*-tests or ANOVA analyses where appropriate, all generated by the GraphPad Prism 8 software (GraphPad Software, La Jolla, CA, USA). In all cases, *p* values < 0.05 were considered significant.

## Figures and Tables

**Table 1:** MerKO mice developed SMG infiltration that increases with age and limited LG involvement. Paraffin-embedded SMG and LG from B6, SjS^S^, and MerKO mice were stained for Hematoxylin and Eosin and observed under a light microscope at 200× for incidence of lymphocytic infiltrates by three independent pathologists.

Strain	Age, Weeks	Submandibular Glands				Lacrimal Glands			
		Number of Mice	Positive			Number of Mice	Positive		
			Number Positive	Percent Positive	Avg # of Infiltrates		Number Positive	Percent Positive	Avg # of Infiltrates
B6	10	6	0	0%	0.0 ± 0.0	3	0	0%	0.0 ± 0.0
SjS^S^	9	7	0	0%	0.0 ± 0.0	7	0	0%	0.0 ± 0.0
MerKO	8	5	0	0%	0.0 ± 0.0	5	0	0%	0.0 ± 0.0
B6	29	6	1	16%	0.3 ± 0.3	4	1	25%	0.5 ± 0.5
SjS^S^	31	5	4	80%	2.0 ± 0.3	5	4	80%	2.4 ± 1.0
MerKO	24	7	4	57%	1.4 ± 0.5	7	2	29%	0.4 ± 0.3
MerKO	32	3	3	100%	3.0 ± 0.5	3	1	33%	0.3 ± 0.3
MerKO	42	4	3	75%	3.0 ± 1.5	4	1	25%	0.5 ± 0.5

**Table 2:** Elevated sMer is correlated to some aspects of severe disease in SjS patients. The relationship between the sMer level and SjS diagnostic criteria was analyzed by Spearman’s rank correlation test where * *p*  <  0.05, and *** *p*  <  0.001. In addition, patient seropositivity for anti-Ro52, Ro60, and La was determined by the Inno-LIA assay.

Parameter	*p* Value	r Value
Focus score	* 0.0419	0.4479
LG vBS score	* 0.0310	0.496
RF (pg/mL)	* 0.0397	0.5315
Anti-Ro60 (pos/equiv/neg)	*** 0.0008	0.7685
Anti-Ro52 (pos/equiv/neg)	0.1524	0.3177
Anti-La (pos/equiv/neg)	0.4773	0.0315
ANA (pg/mL)	0.0879	−0.4077
IgG (pg/mL)	0.0743	0.3929
ESSDAI score	0.0954	0.3572
Schirmer’s test (mm/5 min)	0.3872	−0.0807
WUSF (mL/15 min)	0.1874	−0.2464
ESR (mm/h)	0.1123	0.3609
Age (years)	0.4262	0.0536

## Supplementary Materials

The following are available online at https://www.mdpi.com/article/10.3390/ijms22189711/s1.
