# Supplementary material for: Defective Efferocytosis in a Murine Model of Sjögren’s Syndrome Is Mediated by Dysfunctional Mer Tyrosine Kinase Receptor
Source: Int J Mol Sci. 2021 Sep 8;22(18):9711. doi: 10.3390/ijms22189711 (PMC8466327; doi:10.3390/ijms22189711)
Supplement: Supplementary file 1 [file ijms-22-09711-s001.zip › ijms-1369022-supplementary.pdf]

International Journal of *Molecular Sciences*

MDPI

Article

Defective Efferocytosis in a Murine Model of Sjögren's Syndrome Is Mediated by Dysfunctional Mer Tyrosine Kinase Receptor

Richard Witas, BS[1,2] Astrid Rasmussen, MD, PhD[3], R. Hal Scofield, MD[4,5,6], Lida Radfar, DDS, MS[7], Donald U Stone, MD[8*], Kiely Grundahl, BS[3], David Lewis, DDS[9], Kathy L Sivils, PhD[4*], Christopher J Lessard, PhD[3], A. Darise Farris, PhD[4], Cuong Q Nguyen, PhD[1,2,10]

[1]Department of Infectious Diseases and Immunology, College of Veterinary Medicine, and [2]Department of Oral Biology, College of Dentistry, University of Florida, Gainesville, Florida; [3]Genes and Human Disease Research Program, and [4]Arthritis and Clinical Immunology Research Program, Oklahoma Medical Research Foundation, Oklahoma City, Oklahoma; [5]Department of Internal Medicine, University of Oklahoma Health Sciences Center, Oklahoma City, Oklahoma; [6]Department of Veterans Affairs Medical Center, Oklahoma City, Oklahoma; [7]Department of Oral Diagnosis and Radiology, College of Dentistry, University of Oklahoma Health Sciences Center, Oklahoma City, Oklahoma; [8]Dean McGee Eye Institute, University of Oklahoma Health Sciences Center, Oklahoma City, Oklahoma; [9]Department of Oral Pathology, College of Dentistry, University of Oklahoma Health Sciences Center, Oklahoma City, Oklahoma; [10]Center of Orphaned Autoimmune Diseases, University of Florida, Gainesville, Florida, 32611-0880 USA.

* The current affiliations of these authors are different from their affiliation when they evaluated the study participants. DU Stone is currently at Spokane Eye Clinic, Spokane, Washington. KLS is currently at: Translational Science at The Janssen Pharmaceutical Companies of Johnson & Johnson, Spring House, Pennsylvania, United States

Address correspondence:
Cuong Q. Nguyen, PhD
Department of Infectious Diseases and Immunology
PO Box 110880, College of Veterinary Medicine
University of Florida, Gainesville, Florida 32611-0880 USA
Telephone: 352-294-4180, Fax: 352-392-9704
Email: nguyenc@ufl.edu

Supplementary Materials:

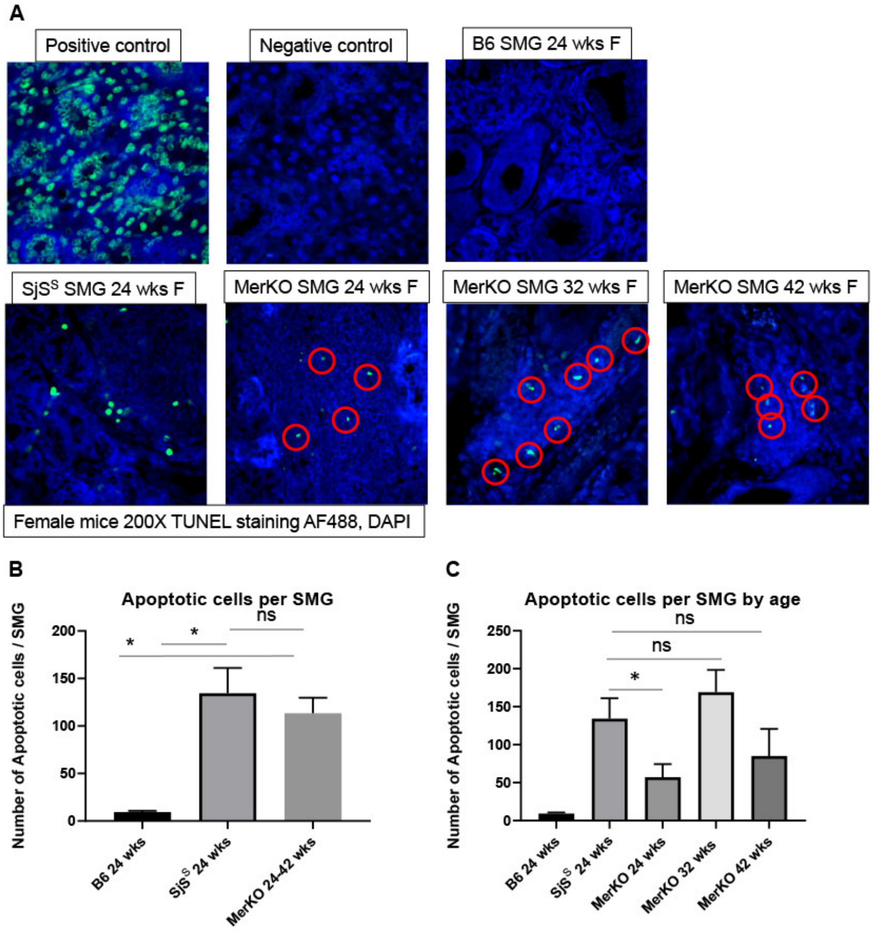

Figure S1. Aged MerKO mice possess elevated apoptotic cells in and around infiltrates. **(A)** Paraffin-embedded SMG sections from B6 (n=3) SjS^S (n=3), and MerKO (n=14) mice underwent TUNEL staining for apoptotic cells (green) and DAPI (blue). **(B, C)** TUNEL stained sections were observed under a Nikon Ti-E fluorescent microscope at 200x and the number of apoptotic cells within each gland was recorded. The statistical significance was calculated by one-way ANOVA where error bars indicate SEM $*p < 0.05$.

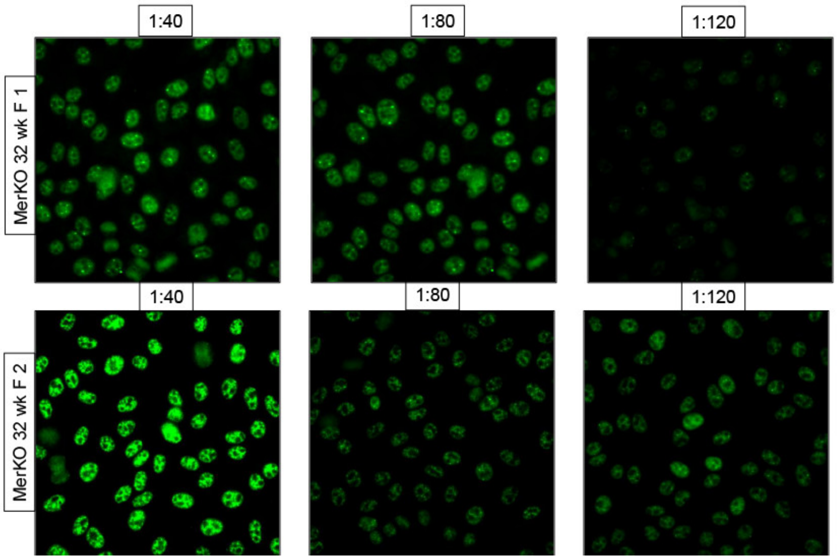

Figure S2. MerKO mice develop robust speckled ANA. MerKO sera was tested for ANAs at dilutions ranging from 1:40 to 1:240 as in Figure 6A.

Table S1. Patient profiles for clinical samples. Abbreviations: primary Sjögren's syndrome (pSjS).

Patient	Age (years)	Sex	Disease	Patient	Age (years)	Sex	Disease
1	28	Female	pSjS	1	33	Female	Non-SjS
2	79	Female	pSjS	2	78	Female	Non-SjS
3	63	Female	pSjS	3	67	Female	Non-SjS
4	71	Female	pSjS	4	68	Female	Non-SjS
5	62	Male	pSjS	5	63	Male	Non-SjS
6	55	Female	pSjS	6	54	Female	Non-SjS
7	46	Female	pSjS	7	48	Female	Non-SjS
8	51	Female	pSjS	8	55	Female	Non-SjS
9	64	Female	pSjS	9	64	Female	Non-SjS
10	30	Female	pSjS	10	30	Female	Non-SjS
11	60	Female	pSjS	11	55	Female	Non-SjS
12	50	Female	pSjS	12	45	Female	Non-SjS
13	58	Female	pSjS	13	62	Female	Non-SjS
14	38	Female	pSjS	14	42	Female	Non-SjS
15	55	Female	pSjS	15	37	Female	Non-SjS
